# Prediction of the Aggressive Clinical Course of Papillary Thyroid Carcinoma Based on Fine Needle Aspiration Biopsy Molecular Testing

**DOI:** 10.3390/ijms25137090

**Published:** 2024-06-28

**Authors:** Sergei A. Lukyanov, Sergei E. Titov, Evgeniya S. Kozorezova, Pavel S. Demenkov, Yulia A. Veryaskina, Denis V. Korotovskii, Tatyana E. Ilyina, Sergey L. Vorobyev, Vladimir A. Zhivotov, Nikita S. Bondarev, Ilya V. Sleptsov, Sergei V. Sergiyko

**Affiliations:** 1 Department of General and Pediatric Surgery, South Ural State Medical University, Chelyabinsk 454092, Russia; 111lll@mail.ru (S.A.L.); korotovskymd@gmail.com (D.V.K.); chlorid@mail.ru (T.E.I.); ssv_1964@mail.ru (S.V.S.); 2 Department of the Structure and Function of Chromosomes, Institute of Molecular and Cellular Biology, SB RAS, Novosibirsk 630090, Russia; microrna@inbox.ru; 3 PCR Laboratory, AO Vector-Best, Novosibirsk 630117, Russia; 4 Department of Natural Sciences, Novosibirsk State University, Novosibirsk 630090, Russia; demps@math.nsc.ru; 5 National Center of Clinical Morphological Diagnostics, Saint Petersburg 192283, Russia; kozorezovaes@yandex.ru (E.S.K.); slvorob@gmail.com (S.L.V.); 6 Institute of Cytology and Genetics, SB RAS, Novosibirsk 630090, Russia; 7 Department of Surgery, National Medical and Surgical Center Named after N.I. Pirogov, Moscow 105203, Russia; opb0321@gmail.com (V.A.Z.); dreamnec@mail.ru (N.S.B.); 8 Department of Faculty Surgery, Saint Petersburg State University, Saint Petersburg 199034, Russia; newsurgery@yandex.ru

**Keywords:** papillary thyroid carcinoma, aggressive variants, recurrence risk, miRNA-221, miRNA-146b, FN1, CDKN2A

## Abstract

Molecular genetic events are among the numerous factors affecting the clinical course of papillary thyroid carcinoma (PTC). Recent studies have demonstrated that aberrant expression of miRNA, as well as different thyroid-related genes, correlate with the aggressive clinical course of PTC and unfavorable treatment outcomes, which opens up new avenues for using them in the personalization of the treatment strategy for patients with PTC. In the present work, our goal was to assess the applicability of molecular markers in the preoperative diagnosis of aggressive variants of papillary thyroid cancer. The molecular genetic profile (expression levels of 34 different markers and BRAF mutations) was studied for 108 cytology specimens collected by fine-needle aspiration biopsy in patients with PTC having different clinical manifestations. Statistically significant differences with adjustment for multiple comparisons (*p* < 0.0015) for clinically aggressive variants of PTC were obtained for four markers: miRNA-146b, miRNA-221, fibronectin 1 (FN1), and cyclin-dependent kinase inhibitor 2A (CDKN2A) genes. A weak statistical correlation (0.0015 < *p* < 0.05) was observed for miRNA-31, -375, -551b, -148b, -125b, mtDNA, CITED1, TPO, HMGA2, CLU, NIS, SERPINA1, TFF3, and TMPRSS4. The recurrence risk of papillary thyroid carcinoma can be preoperatively predicted using miRNA-221, FN1, and CDKN2A genes.

## 1. Introduction

Surgery is the main treatment modality for papillary thyroid carcinoma (PTC). The extent of primary surgery depends on several clinical characteristics of the disease, such as tumor size, metastases, and macroscopic extrathyroidal extension, as well as the presence of microscopic vascular invasion and the morphological subtype of PTC [1].

When tumor size is < 4.0 cm and neither regional nor distant metastases are present, patient survival is independent of the extent of the surgery (total thyroidectomy or hemithyroidectomy) [2]. Most National Comprehensive Cancer Network (NCCN) committee members recommend performing total thyroidectomy for thyroid cancer patients having the following clinical data: T3 or T4; cytologically verified N1; the presence of M1; aggressive morphological subtypes; a history of significant exposure to radiation within the head and neck area; and a family history of cancer. The authors of the guidelines advocate for unilateral hemithyroidectomy in the group of patients having a low recurrence risk as the final treatment modality for most patients with papillary thyroid carcinoma because of low mortality and recurrence rate as well as a higher rate of complications related to thyroidectomy [3].

In some cases, it is difficult to plan surgery to an extent that would comply with the ATA and NCCN guidelines because of the lack of data on the morphological subtype of PTC in the cytology report. Furthermore, the potentially aggressive PTC could have possibly been diagnosed early and did not have time to manifest itself as extrathyroidal extension and vascular invasion, while no regional lymph node involvement was detected because of the microscopic size of the metastases. Total thyroidectomy with central lymph node dissection followed by radioactive iodine therapy would be preferred in this case [4]. Furthermore, for some patients with PTC at the preoperative stage, the cytology report specifies “follicular neoplasm” (Bethesda category IV) or “atypia of undetermined significance” (Bethesda category III) [5,6]. As a result, reoperation may be needed if hemithyroidectomy is chosen as the primary surgical treatment option. Molecular testing can be used to refine indications for surgical treatment in patients with vague cytological findings [7]. The application of molecular research in patients preoperatively diagnosed with PTC for personalizing the treatment approach remains poorly investigated.

Somatic mutations currently are the best-studied molecular markers of PTC aggressiveness. It has long been observed that early genetic events of thyroid cancer progression (e.g., BRAF mutations) are frequently found in patients with both well-differentiated thyroid cancer and poorly differentiated or anaplastic thyroid cancer because they are involved in the initiation of tumor development. In contrast, late genetic events (e.g., TP53 gene mutations) are more common in tumors that gradually lose thyroid differentiation and are therefore associated with tumor progression and an unfavorable outcome [8]. In patients with thyroid cancer, TERT promoter mutations are classified as a late event and are found in more aggressive thyroid cancers, being more common in patients with poorly differentiated or anaplastic cancer (up to ~70% of cases) than those with well-differentiated PTC and follicular thyroid carcinoma (FTC) (~10–20%). Moreover, TERT mutations have been recognized as an independent predictor of tumor recurrence, distant metastases, poor prognosis, and mortality in patients with well-differentiated PTC and FTC [9].

Attempts at recurrence risk stratification based on associations between molecular changes and the risk of disease aggressiveness (mostly the risk of developing distant metastases) have been reported in many studies [10,11,12]. The researchers have formed molecular risk groups (MRGs) the low-risk MRG involved RAS-like changes, and the intermediate-risk MRG involved BRAF-like changes. The high-risk profile involved TERT, TP53, AKT1, and PIK3CA mutations. Studies have demonstrated that the molecular profile can predict the risk of developing distant metastases rather accurately [13,14] and generally increase the accuracy of recurrence risk assessment according to the ATA risk stratification system [15]. However, this model cannot be used to predict other signs of aggressiveness of PTC such as the emergence of metastases in cervical lymph nodes and extrathyroidal extension.

As for other types of molecular markers (assessment of expression of miRNA and various genes involved in thyroid function), the feasibility of using them to predict the development of PTC has been studied by different researchers; however, none of the new markers has become widely recognized [16,17,18].

In our previous studies, we investigated the diagnostic potential of several types of molecular markers: BRAF V600E mutation, and the relative expression of miRNA and protein-coding genes, as well as the mitochondrial/nuclear DNA ratio for preoperative detection of thyroid cancer. Some of the results of these studies have already been published [19]. In this work, the preoperative prognostic potential of certain markers for PTC was investigated. We analyzed 34 molecular markers in cytology specimens collected by fine needle aspiration biopsy and assessed the correlation between them, as well as the clinical and morphological features of tumors and the risk of PTC recurrence.

## 2. Results

Table 1 summarizes the clinical characteristics of 108 patients with PTC enrolled in the study.

Among 108 patients with PTC, there were 86 (79.6%) females and 22 (20.4%) males. The median age at diagnosis was 47.5 (37–60.25) years (the youngest patient was 22 years old; the oldest one was 85 years old). No differences in sex ratio were observed in the groups being compared. The minimum and maximum tumor diameter was 0.6 cm and 8.0 cm, respectively. The tumor was multifocal in 61 (56.5%) cases; vascular invasion was observed in 58 (53.4%) cases. Twenty-five (23.1%) patients had a macroscopic extrathyroidal extension. Metastatic spread to cervical lymph nodes was detected in 53 (49.1%) cases. In accordance with the 2015 ATA risk stratification system, 23 (21.3%) patients were categorized into the low-risk group; 60 (55.6%), into the intermediate-risk group; and 25 (23.1%) patients into the high-risk group.

An intergroup comparative analysis of the association between the clinical/pathological characteristics of PTC and BRAF V600 mutations was carried out (Table 2).

Thus, for BRAF mutations, no statistically significant association was found for sex, multifocality, extrathyroidal extension, metastasis, or vascular invasion. The BRAF mutations were statistically significantly more frequent in the groups of patients with intermediate (80%, *p* = 0.03) and high (84%, *p* = 0.04) ATA recurrence risk compared to the low-risk group (56.5%).

The correlation between tumor size in patients with PTC and the expression level of the miRNA and genes being studied was analyzed. With adjustment for multiple comparisons, a statistically significant correlation level among 34 parameters was achieved only for the expression level of the TPO gene (*p* = 0.00045, Spearman’s rank coefficient −0.33). The correlation between tumor size and the TPO level was negative and relatively strong: the larger the tumor size, the lower the expression level of the thyroid peroxidase gene. There was also a correlation between the expression of the SLC26A7 gene (*p* = 0.005, Spearman’s rank coefficient −0.26) and the mtDNA level (*p* = 0.006, Spearman’s rank coefficient 0.26). However, the required confidence level *p* < 0.0015 was not achieved for these markers.

The relative expression levels of 11 miRNAs, the mtDNA level, and the expression of 22 genes were compared. The results are summarized in Table 3.

A comparison of the expression of molecular markers revealed differences in all the clinical/morphological groups except for the group of patients having tumors characterized by vascular invasion; a weak correlation was also detected in the case of the multifocal nature of cancer. No statistical significance (*p* > 0.05) was observed for miRNA: -199b, -223, -451a, and -21. Among the studied genes, we found no correlation with clinical manifestations of PTC for the following markers: TSHR (the TSH receptor gene playing a pivotal role in controlling the metabolism of thyrocytes) and SLC26A7 (codes for iodine receptor), whose expression is significantly downregulated in patients with anaplastic thyroid cancer [20]; CPQ (carboxypeptidase that plays a certain role in the release of the thyroxine hormone from its precursor, thyroglobulin) and RXRG (retinoic acid receptor), whose expression was found to decrease in patients with follicular thyroid cancer [21,22]; SPATA18 (the key regulator of mitochondrial quality), playing a role in the development of oncocytic cell carcinoma of the thyroid [23]; APOE (apolipoprotein, a protein involved in lipid transport between organelles through plasma and interstitial fluids); ASF1B (a histone chaperone) and TIMP1 (a metalloproteinase inhibitor acting as a growth factor), with a reduction in the overall survival in patients with thyroid cancer [24,25]; AFAP1L2 (transcriptional activator), whose upregulated transcription is associated with thyrocyte apoptosis [26]; ECM1 (extracellular matrix protein stimulating the proliferation of endothelial cells and promoting angiogenesis), whose expression considered to be decreased in patients with advanced forms of PTC [27]; and DIO1 (iodothyronine deiodinase, which is responsible for the deiodination of T4 to T3 and T3 to T2), which is differently expressed in patients with follicular adenoma and thyroid carcinoma [28].

A weak statistical correlation (0.0015 < *p* < 0.05) was observed for miRNA-31, -375, -551b, -148b, -125b, for mtDNA, and for the following genes:

- The TPO (thyroid peroxidase) gene: the decline in its expression is associated with resistance to radioactive iodine therapy [29]. We detected that TPO expression depends on tumor size while being weakly associated with extrathyroidal invasion (*p* = 0.01) and a high/low recurrence risk (*p* = 0.02). Taking into account the fact that radioactive iodine-resistant tumors are more likely to be large-sized, it appears that thyroid peroxidase activity is its consequence rather than a cause.
- The CITED1 gene, which is associated with the development of follicular cancer [28]. Differences were observed for such parameters as cervical lymph node metastases (*p* = 0.01), extrathyroidal extension (*p* = 0.02), and high/low recurrence risk (*p* = 0.004).
- The HMGA2 gene: expression of this gene is believed to be associated with lymphogenic metastasis and vascular invasion [30]. According to our data, weak differences were observed for the groups of patients with/without metastases (*p* = 0.02), with/without extrathyroidal extension (*p* = 0.01), with/without vascular invasion (*p* = 0.05), and with moderate/high recurrence risk (*p* = 0.01).
- The NIS (sodium/iodine symporter) gene, whose expression level is reduced in most thyroid carcinomas [31]. Differences were observed for the groups of patients with a low/high (*p* = 0.05) and moderate/high (*p* = 0.0049) recurrence risk.
- The CLU gene (clusterin alpha chain, an extracellular chaperone preventing the aggregation of non-native proteins) whose upregulated expression is associated with better survival prognosis [24]. Differences were observed in groups of patients with/without metastases (*p* = 0.005) and multifocal/unifocal cancer (*p* = 0.01).
- The SERPINA1 (serine protease inhibitor) gene: its association with the stage and the multifocal nature of thyroid cancer has been reported [32]. Differences were observed for the groups of patients with/without metastases (*p* = 0.004).
- The TFF3 gene: its downregulated expression was observed in patients with follicular thyroid cancer [21]. Differences were detected in the groups with/without metastases (*p* = 0.02), with/without extrathyroidal extension (*p* = 0.002), and with a high/low (*p* = 0.01) and moderate/high risk (*p* = 0.003).
- The TMPRSS4 (transmembrane serine protease) gene is characterized by increased expression in patients with PTC [27]. Differences in groups of patients with/without metastases (*p* = 0.04), and with a low/intermediate (*p* = 0.05) and low/high (*p* = 0.01) recurrence risk.

**Figure 1:**
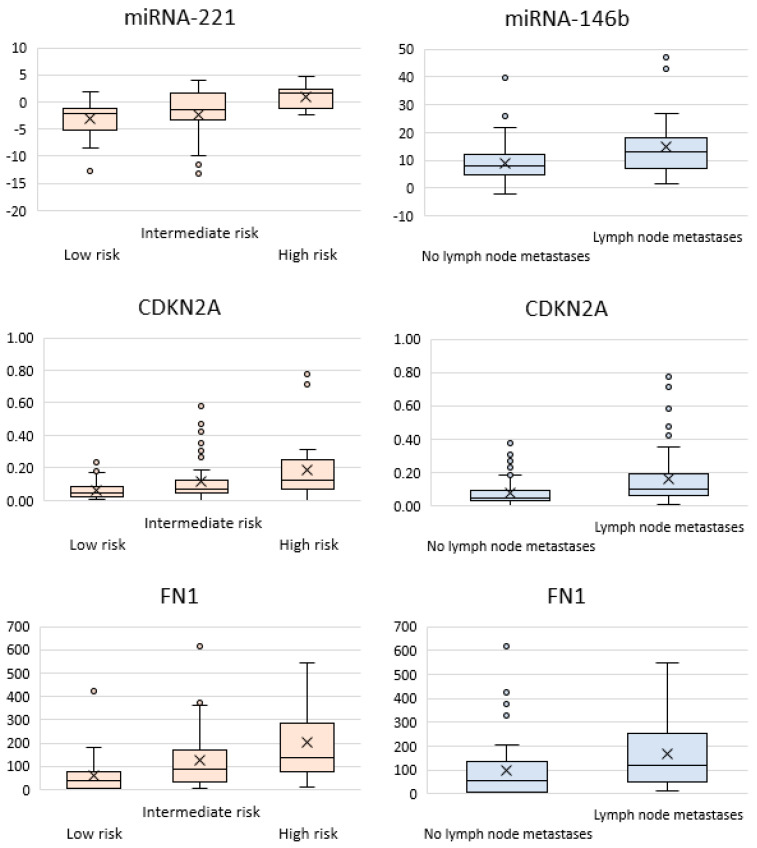
Box–whisker plots for the relative expression level of selected miRNAs (miR-146b, miR-221) and genes (FN1 and CDKN2A). Inner line, the median value; cross, the mean value; box, upper and lower quartiles; whisker, non-outlier range; circles, outliers. The data are presented for samples belonging to the low/intermediate/high ATA recurrence risk groups or to the groups with/without lymph node metastases.

Statistically significant differences with adjustment for multiple comparisons (*p* < 0.0015) were obtained for four markers: miR-146b, miR-221, FN1, and CDKN2A (Figure 1).

The miRNA-146b level was on average 1.7-fold higher in tumors with regional metastases to cervical lymph nodes than without them (*p* = 0.0003). The miRNA-221 level was 1.9-fold higher in patients with extrathyroidal extension (*p* = 0.00005), as well as 3.4-fold and 2-fold higher in the groups of patients with a high and intermediate recurrence risk compared to the low-risk groups (*p* = 0.000013 and *p* = 0.001, respectively). An upregulated expression was observed for the FN1 (fibronectin, which is usually believed to be associated with the development of follicular cancer [28]) gene in groups of patients having cervical lymph node metastases (1.7-fold higher; *p* = 0.0004) and with a high and intermediate ATA recurrence risk (3.75-fold and 1.6-fold; *p* = 0.000013 and *p* = 0.001, respectively). The expression of the CDKN2A (cyclin-dependent kinase inhibitor 2A, which is associated with anaplastic thyroid cancer [33]) gene was twice higher in patients with cervical lymph node metastases (*p* = 0.00014) and 3.3-fold higher in patients having a high recurrence risk than those having a low risk (*p* = 0.0012).

**Figure 2:**
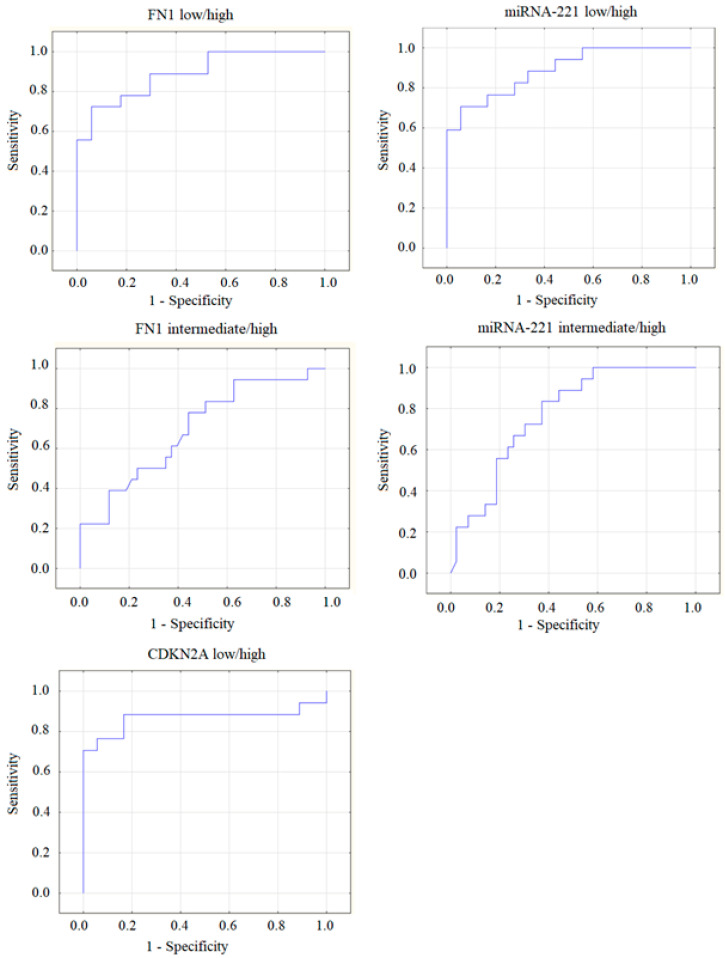
ROC curves for the three best markers that allow identifying samples with high ATA recurrence risk: miRNA-221, FN1, and CDKN2A genes. The curves are shown for comparison of either low versus high, or intermediate versus high ATA recurrence risk groups.

Hence, differences in recurrence risk were detected only for 3 out of the 34 studied molecular genetic factors: expression of miRNA-221, as well as the FN1 and CDKN2A genes. The areas under the ROC curves (AUC) were calculated for these markers (Figure 2).

The highest ROC AUC values were obtained for miRNA-221, between the low-/high-risk (AUC = 0.88) and the intermediate-/high-risk (AUC = 0.77) groups; for CDKN2A, the AUC between the low-/high-risk group was 0.87; and for FN1, the AUC between the low-/high-risk group was 0.81 and between the intermediate-/high-risk group, AUC = 0.69.

The calculated values of these markers gave grounds for categorizing a patient into the group of high recurrence risk according to ATA guidelines with a high sensitivity. For the CDKN2A gene, at a cut-off value of 0.044, the sensitivity was 88.5% (95% CI: 69.8–97.5); and the specificity was 47.8% (95% CI: 26.8–69.4). For the FN1 gene, at a cut-off value of 8.5, the sensitivity was 100% (95% CI: 86.7–100); and the specificity was 43.5% (95% CI: 23.2–65.5). The expression level of miRNA-221 > 0.47 has a 96.2% (95% CI: 80.4–99.9) prognostic sensitivity for high recurrence risk and a specificity of 60.9% (95% CI: 38.5–80.3).

## 3. Discussion

Timely adequate surgical treatment ensures good five-year survival in most patients with PTC; metastatic spread to regional cervical lymph nodes or local cancer recurrence is detected after primary surgery in 20% of cases [34,35]. More accurate risk stratification of PTC recurrence is needed to avoid overtreatment of the majority of patients having a “favorable” prognosis and to ensure adequate treatment to the minority having an “aggressive” type of carcinoma.

Out of the set of molecular genetic events in patients with PTC, the ATA guidelines (2015) recommend using only the BRAF and TERT mutations as markers of cancer with the least favorable prognosis [1]. However, later reviews of this strategy yielded controversial conclusions. Some studies demonstrated that the BRAF mutation was associated with an extrathyroidal extension of the tumor and metastatic spread to cervical lymph nodes [34], while another study did not reveal this association [35]. In our work, the BRAF mutations were more frequent in groups of patients with intermediate and high recurrence risk according to ATA; however, statistical differences were minimal.

It is still extremely important to further search for efficient markers to perform accurate stratification of PTC recurrence [6,36]. MicroRNAs can be such markers; a large number of them, both oncogenic and tumor suppressor ones, have already been identified [37]. In these studies, different miRNAs were shown to correlate with signs of tumor aggressiveness such as extrathyroidal extension, metastatic spread to lymph nodes, distant metastases, and disease recurrence. We examined 11 different miRNAs in preoperative cytology specimens and demonstrated that expression levels of two of them (miRNA-146b and miRNA-221) were significantly increased in the subgroups of patients having such features as regional metastases and extrathyroidal extension. The present work confirmed the findings of our previous study, where the expression levels of these same miRNAs were evaluated using postoperative specimens, and the high risk of PTC recurrence was 9.7 times more probable (95% CI 3.1–29.5) if the miR-221 level was >1.0 [38].

The genes responsible for various processes occurring in thyrocytes, whose activity can be determined according to their expression level, can be other candidates to act as molecular genetic markers. We can mention two out of twenty-two such genes that have been studied: the FN1 (fibronectin) and CDKN2A (cyclin-dependent kinase inhibitor 2A) genes. According to our findings, they turned out to be associated with the metastatic spread to lymph nodes and a higher recurrence risk.

For assessing the recurrence risk, one needs to understand that some patients having potentially aggressive PTC variants could be categorized into the low-risk group because of early tumor detection. However, the molecular markers may be indicative of their aggressiveness even before regional metastases and extrathyroidal extension develop. Therefore, one of the study objectives was quantifying the expression of molecular genetic markers, which can be used to preoperatively predict the high risk of PTC recurrence with nearly 100% sensitivity, while specificity is not necessarily high.

These markers were as follows: a relative expression of the CDKN2A gene > 0.044 (88.5% sensitivity); a relative expression of the FN1 gene > 8.5 (100% sensitivity); and a relative miRNA-221 expression > 0.47 (96.2% sensitivity).

## 4. Materials and Methods

### 4.1. Clinical Material 

The study involved 125 patients with PTC who had been operated on at three sites (the clinical setting of the Division of General and Pediatric Surgery (Chelyabinsk, Russia), the Department of Surgery at the Pirogov National Medical and Surgical Center (Moscow, Russia), and the Pirogov Clinic of High Medical Technologies at St. Petersburg State University (St. Petersburg, Russia)) in 2022–2023. The study included patients who had undergone total thyroidectomy. Lymph node dissection was performed for 53 of them. Central neck compartment lymph node dissection (level VI-VII) was performed for 36 of them, and central and lateral neck compartment lymph node dissection (level II-VII) was performed for 17 patients.

The clinical data and risk stratification (2015 ATA risk stratification system) were performed by analyzing patients’ medical records. Histology and cytology examinations were conducted by residential morphologists in the respective clinical settings. All the specimens were subsequently re-examined by two independent morphologists working at the National Center for Clinical Morphological Diagnostics (St. Petersburg, Russia); reports for cytology specimens were created in compliance with the 2023 Bethesda System; for histology specimens, reports were created in compliance with the International Histological Classification of Thyroid Tumors (5th Edition, 2022) [35]. After paucicellular cytology specimens and insufficiently informative histology specimens had been discarded, a total of 108 patients remained in the study. Therefore, all the findings obtained in this study refer to papillary thyroid cancer only rather than to the entire group of differentiated thyroid cancers.

### 4.2. Choosing the Set of Molecular Markers

The primary set of mRNA for analysis was selected according to the available literature. Protein-coding genes were chosen so that their exon–intron structure enabled the detection of mRNA without preliminary purification to remove genomic DNA. The mRNA list comprised 22 genes: FN1, Geminin (GMNN), CDKN2A, TIMP1, CITED1, TPO, SLC26A7, HMGA2, CPQ, RXRG, SPATA18, APOE, ASF1B, AFAP1L2, CLU, ECM1, DIO1, NIS, SERPINA1, TFF3, TMPRSS4, and TSHR.

The set of miRNAs was selected according to our own data [19] and analysis of the literature data; a total of 11 miRNAs were involved in the experimental analysis: miR-146b-5p, miR-199b-5p, miR-221-3p, miR-223-3p, miR-31-5p, miR-375, miR-451a, miR-551b-3p, miR-148b-3p, miR-21-5p, and miR-125b-5p.

The mtDNA/nDNA ratio was used as a criterion for the presence of oncocytic cells in the clinical specimen [19]. Hence, a total of 34 molecular genetic markers have been analyzed in this study. The presence of the somatic BRAF V600E mutation was investigated separately.

### 4.3. Total Nucleic Acid Extraction

The nucleic acids were extracted from FNAC preparations as described in ref. [39]: The dried cytological preparation was washed into a 1.5 mL microcentrifuge tube with three 200 μL portions of guanidine lysis buffer (4 M guanidine isothiocyanate, 25 mM sodium citrate pH 7.0, 0.3% sarcosyl, and 0.1% 2-mercaptoethanol). The sample was vigorously mixed and incubated in a thermal shaker for 15 min at 65 °C. Next, an equal volume of isopropanol was added. The reaction solution was thoroughly mixed and kept at room temperature for 5 min. After centrifugation for 15 min at 14,000× *g*, the supernatant was discarded, and the pellet was washed with 500 μL of 70% ethanol and 300 μL of acetone. Finally, the RNA was dissolved in 200 μL of deionized water. If not analyzed immediately, RNA samples were stored at −20 °C until further use.

### 4.4. Semi-Quantification of Messenger RNA Level 

A semi-quantitative assessment of the mRNA level was performed by real-time RT-PCR with specific primers and fluorescently labeled probes for detecting mRNA of the respective genes and the housekeeping gene PGK1 (phosphoglycerate kinase), which is used as a normalization gene. The RT-PCR protocol was as follows: incubation at 45 °C—30 min; heating at 95 °C—2 min, 50 cycles: denaturation at 94 °C—10 s; annealing and extension: 60 °C—20 s [19]. The relative expression level was calculated using the 2^−ΔCq^ method [40]. All oligonucleotides used in the work are listed in Table S1.

### 4.5. MicroRNA Detection 

The detection of 11 miRNAs was conducted by stem-loop PCR [41]. Reverse transcription (RT) followed by real-time PCR was conducted individually for each miRNA in compliance with the procedure described in ref. [19]. The RT reaction mix contained 3 μL of RNA preparation (4–47 ng/μL; 15 ng/μL on average), 21.6% trehalose, RT buffer (50 mM Tris-HCl, pH 8.3, 75 mM KCl, 3 mM MgCl2), 0.4 mM of each dNTP, 1% BSA, 100U M-MLV reverse transcriptase, and 0.2 μM of an appropriate RT primer. The RT reaction was incubated for 30 min at 42 °C, which was followed by heat inactivation for 2 min at 95 °C. In total, 3 μL of RT mix was used per one PCR reaction. Real-time PCR was performed using a CFX96 thermal cycler (Bio-Rad Laboratories, Hercules, California, USA). The total volume of each reaction was 30 μL and encompassed 3 μL of cDNA, PCR buffer (50 mM Tris-HCl pH 8.9, 1.5 mM MgCl2, 25 mM KCl, 0.1% Triton X-100), 0.4 mM of each dNTP, 1% BSA, 1U Taq polymerase (SibEnzyme, Novosibirsk, Russia) pre-mixed with center-specific monoclonal antibodies (Takara Bio USA, Inc., Mountain View, CA, USA), 0.5 units of uracil-DNA glycosylase (SibEnzyme, Novosibirsk, Russia), 0.5 μM of each primer, and 0.25 μM of Taqman probe. Real-time PCR cycling conditions were as follows: 2 min UDG incubation at 50 °C and the pre-denaturation step at 94 °C for 2 min, followed by 50 cycles of denaturation (94 °C for 10 s), annealing, and elongation (60 °C for 20 s). A single-replicate analysis was performed for each specimen. The miRNA level was normalized to the geometric mean of the levels of three reference miRNAs (miR-197-3p, -23a-3p, and -29b-3p) using the 2^−ΔCq^ method. All oligonucleotides used in the work are listed in Table S1.

### 4.6. Quantification of the Ratio between the Mitochondrial and Nuclear DNA Copy Number (the mtDNA/nDNA Ratio) 

Mitochondrial and nuclear DNA were detected by real-time PCR. Real-time PCR was performed using a CFX96 thermal cycler with the same composition of the reaction mixture as described above. The PCR protocol was as follows: pre-heating at 95 °C—2 min, 50 cycles: denaturation at 94 °C—10 s; annealing and extension at 60 °C—20 s [19]. The ratio was determined using the 2^−ΔCq^ method.

### 4.7. Detection of Somatic BRAF Mutation 

All the samples were tested for somatic mutations V600E, V600E2, and V600K in the BRAF gene. Somatic mutations were detected using allele-specific PCR with the same composition of the reaction mixture as described above. The PCR protocol was as follows: pre-heating at 95 °C—2 min, 50 cycles; denaturation at 94 °C—10 s; and annealing and extension at 60 °C—15 s [19].

### 4.8. Statistical Data Analysis

Statistical data analysis was conducted using the SPSS Statistics 23 (IBM, Armonk, NY, USA) and Excel software (Microsoft Office 2019, Redmond, WA, USA). The data are presented as the mean and median values, Q1 and Q3. All the statistical analyses were conducted using the Mann–Whitney test to compare the two groups. The family-wise error rate (FWER) assessed using the Bonferroni method was used to solve the multiple hypothesis testing problem. The significance level *p* was calculated as 0.05 divided by the number of parameters being compared. In our study, we compared 34 parameters, so *p* < 0.05/34 = 0.0015 was considered statistically significant. The correlation between tumor size and miRNA and gene expression levels was evaluated using Spearman’s rank correlation coefficient. The association between the BRAF mutation status and each clinical pathological variable was assessed using Pearson’s chi-squared test (χ^2^) and Fisher’s exact test when the number of patients was <5. If *p* < 0.05, the difference was considered statistically significant. The odds ratio (OR) was determined by univariate analysis; 95% confidence intervals were also calculated.

Binary classification involving plotting the ROC curves was used for the objective evaluation of the predictive power for the expression levels of different miRNAs and genes to predict the risk of PTC recurrence. The ROC curve shows the dependence between the number of correctly classified positive examples (a true positive set) on the number of incorrectly classified negative examples (a false negative set). The assessed reliability score is expressed as sensitivity and specificity parameters. The tests were compared with allowance for the area under the ROC curves (AUC). It is fair to say with some assumptions that the closer the AUC parameter to unity, the higher the predictive power of a test is. The following expert scale was employed for the AUC values (it can be used to assess the quality of a model): 0.9–1.0—excellent; 0.8–0.9—very good; 0.7–0.8—good; 0.6–0.7—moderate; and 0.5–0.6—poor.

## 5. Conclusions

Identifying novel molecular markers for predicting aggressiveness and the risk of PTC recurrence is a very important problem to be solved since it will allow for reducing the surgery extent and, therefore, the number of potential postoperative complications on the one hand. On the other hand, more extensive surgical interventions, even in patients from the low recurrence risk group but having a high expression of these markers, will help reduce the future risk of tumor recurrence. A detailed understanding of the potential risks of PTC recurrence at the treatment planning stage will help ensure personalized therapy and will therefore improve patients’ quality of life and treatment outcomes.

## Figures and Tables

**Table 1:** Clinical characteristics of patients with PTC.

Characteristic	N (%)
Median age (Q1–Q3)	47.5 (37–60.3)
Sex ratio (male/female)	22/86
Metastases in central lymph nodes	28 (26%)
Metastases in lateral lymph nodes	25 (23.1%)
Multifocal nature	61 (56.5%)
Extrathyroidal extension (macroscopic invasion)	25 (23.1%)
Vascular invasion	58 (53.4%)
**Variants of PTC**	
Classical	35 (32.4%)
Oncocytic	30 (27.8%)
Tall cell	19 (17.6%)
Follicular	15 (13.9%)
Warthin-like	5 (4.6%)
Solid	4 (3.7%)
**ATA risk stratification**	
Low risk	23 (21.3%)
Intermediate risk	60 (55.6%)
High risk	25 (23.1%)

**Table 2:** Analysis of the association between clinical/pathological characteristics of PTC and BRAFV660E mutation.

Parameter	Total Number	BRAF Mutations		Odds Ratio (95% CI)	*p*
		yes	no		
**Sex**					
females	86	64	22	0.84 (0.26–2.76)	0.78
males	22	18	4		
**Multifocal nature**					
unifocal	47	35	12	0.86 (0.35–2.1)	0.75
multifocal	61	47	14		
**Extrathyroidal extension**					
no	25	21	4	0.52 (0.16–1.71)	0.28
yes	83	61	22		
**Metastases to the cervical lymph nodes**					
no	55	39	16	0.56 (0.23–1.39)	0.21
yes	53	43	10		
**Vascular invasion**					
no	50	34	16	0.44 (0.17–1.09)	0.07
yes	58	48	10		
**ATA recurrence risk**					
low	23	13	10	low/intermediate 0.32 (0.11–0.91)	**0.03**
intermediate	60	48	12	intermediate/high 0.76 (0.22–2.6)	0.66
high	25	21	4	low/high 0.24 (0.06–0.95)	**0.04**

Significant differences (*p* < 0.05) are shown in bold.

**Table 3:** The *p* value in the groups of patients with PTC being compared.

**Group**	**miR-146b**	**miR-199b**	**miR-221**	**miR-223**	**miR-31**	**miR-375**
Metastases to cervical lymph nodes	**0.0003**	0.84	0.01	0.05	0.04	0.14
Extrathyroidal extension	0.15	0.23	**0.00006**	0.38	0.01	0.02
Vascular invasion	0.38	0.24	0.58	0.98	0.28	0.09
Multifocal nature	0.12	0.39	0.84	0.04	0.18	0.95
Low/intermediate	0.04	0.60	0.06	0.16	0.87	0.1
Low/high	0.02	0.59	**0.00001**	0.10	0.09	0.007
Intermediate/high	0.4	0.18	**0.001**	0.6	0.01	0.08
**Group**	**miR-451a**	**miR-551b**	**miR-148b**	**miR-21**	**miR-125b**	**mtDNA**
Metastases to cervical lymph nodes	0.20	0.07	0.04	0.41	0.34	0.01
Extrathyroidal extension	0.55	0.01	0.90	0.95	0.03	0.58
Vascular invasion	0.93	0.22	0.70	0.26	0.63	0.05
Multifocal nature	0.26	0.21	0.005	0.48	0.89	0.01
Low/intermediate	0.18	0.20	0.07	0.17	0.98	0.004
Low/high	0.15	0.01	0.18	0.50	0.11	0.01
Intermediate/high	0.91	0.02	0.65	0.70	0.03	0.72
**Group**	**FN1**	**GMNN**	**CDKN2A**	**TIMP1**	**CITED1**	**TPO**
Metastases to cervical lymph nodes	**0.0004**	0.25	**0.00015**	0.05	0.01	0.09
Extrathyroidal extension	0.002	0.85	0.003	0.27	0.02	0.02
Vascular invasion	0.33	0.26	0.24	0.83	0.63	0.28
Multifocal nature	0.11	0.74	0.05	0.12	0.09	0.57
Low/intermediate	**0.001**	0.71	0.03	0.38	0.09	0.12
Low/high	**0.00006**	0.88	**0.0012**	0.20	0.004	0.02
Intermediate/high	0.03	0.74	0.01	0.40	0.08	0.06
**Group**	**SLC26A7**	**HMGA2**	**CPQ**	**RXRG**	**SPATA18**	**APOE**
Metastases to cervical lymph nodes	0.53	0.02	0.59	0.46	0.05	0.54
Extrathyroidal extension	0.06	0.01	0.81	0.65	0.82	0.12
Vascular invasion	0.64	0.05	0.35	0.7	0.10	0.53
Multifocal nature	0.69	0.61	0.47	0.84	0.14	0.80
Low/intermediate	0.96	0.71	0.05	0.34	0.35	0.13
Low/high	0.28	0.18	0.22	0.81	0.50	0.05
Intermediate/high	0.05	0.01	0.38	0.48	0.99	0.25
**Group**	**ASF1B**	**AFAP1L2**	**CLU**	**ECM1**	**DIO1**	**NIS**
Metastases to cervical lymph nodes	0.40	0.82	0.005	0.91	0.61	0.07
Extrathyroidal extension	0.27	0.73	0.80	0.54	0.43	0.005
Vascular invasion	0.15	0.17	0.47	0.88	0.61	0.81
Multifocal nature	0.94	0.99	0.01	0.17	0.98	0.06
Low/intermediate	0.05	0.91	0.28	0.48	0.49	0.82
Low/high	0.74	0.81	0.56	0.86	0.38	0.05
Intermediate/high	0.11	0.73	0.55	0.47	0.53	0.0049
**Group**	**SERPINA1**	**TFF3**	**TMPRSS4**	**TSHR**		
Metastases to cervical lymph nodes	0.004	0.02	0.04	0.39		
Extrathyroidal extension	0.47	0.002	0.12	0.48		
Vascular invasion	0.28	0.84	0.07	0.81		
Multifocal nature	0.19	0.18	0.78	0.05		
Low/intermediate	0.04	0.34	0.05	0.31		
Low/high	0.09	0.01	0.01	0.21		
Intermediate/high	0.8	0.003	0.36	0.73		

Significant differences (*p* < 0.0015) are shown in bold.

## Data Availability

The raw data supporting the conclusions of this article will be made available by the authors upon request.

## Supplementary Materials

The following supporting information can be downloaded at: https://www.mdpi.com/article/10.3390/ijms25137090/s1.

## References

[1] Haugen B.R., Alexander E.K., Bible K.C., Doherty G.M., Mandel S.J., Nikiforov Y.E., Pacini F., Randolph G.W., Sawka A.M., Schlumberger M. (2016). 2015 American Thyroid Association Management Guidelines for Adult Patients with Thyroid Nodules and Differentiated Thyroid Cancer: The American Thyroid Association Guidelines Task Force on Thyroid Nodules and Differentiated Thyroid Cancer. Thyroid.

[2] Davies L., Welch H.G. (2010). Thyroid cancer survival in the United States: Observational data from 1973 to 2005. Arch. Otolaryngol. Head Neck Surg..

[3] Haddad R.I., Bischoff L., Ball D., Bernet V., Blomain E., Busaidy N.L., Campbell M., Dickson P., Duh Q., Ehya H. (2022). Thyroid Carcinoma, Version 2.2022, NCCN Clinical Practice Guidelines in Oncology. J. Natl. Compr. Cancer Netw..

[4] Sun J.H., Li Y.R., Chang K.H., Liou M.J., Lin S.F., Tsai S.S., Yu M.C., Hsueh C., Chen S.T. (2022). Evaluation of recurrence risk in patients with papillary thyroid cancer through tumor-node-metastasis staging: A single-center observational study in Taiwan. Biomed. J..

[5] Patel K.N., Angell T.E., Babiarz J., Barth N.M., Blevins T., Duh Q.Y., Ghossein R.A., Harrell R.M., Huang J., Kennedy G.C. (2018). Performance of a genomic sequencing classifier for the preoperative diagnosis of cytologically indeterminate thyroid nodules. JAMA Surg..

[6] Valderrabano P., Leon M.E., Centeno B.A., Otto K.J., Khazai L., McCaffrey J.C., Russell J.S., McIver B. (2016). Institutional prevalence of malignancy of indeterminate thyroid cytology is necessary but insufficient to accurately interpret molecular marker tests. Eur. J. Endocrinol..

[7] Patel K.N., Yip L., Lubitz C.C., Grubbs E.G., Miller B.S., Shen W., Angelos P., Chen H., Doherty G.M., Fahey T.J. (2020). The American Association of Endocrine Surgeons Guidelines for the definitive surgical management of thyroid disease in adults. Ann. Surg..

[8] Panebianco F., Nikitski A.V., Nikiforova M.N., Nikiforov Y.E. (2019). Spectrum of TERT promoter mutations and mechanisms of activation in thyroid cancer. Cancer Med..

[9] Melo M., da Rocha A.G., Vinagre J., Batista R., Peixoto J., Tavares C., Celestino R., Almeida A., Salgado C., Eloy C. (2014). TERT promoter mutations are a major indicator of poor outcome in differentiated thyroid carcinomas. J. Clin. Endocrinol. Metab..

[10] Patel S.G., Carty S.E., McCoy K.L., Ohori N.P., LeBeau S.O., Seethala R.R., Nikiforova M.N., Nikiforov Y.E., Yip L. (2017). Preoperative detection of RAS mutation may guide extent of thyroidectomy. Surgery.

[11] Song Y.S., Lim J.A., Choi H., Won J.K., Moon J.H., Cho S.W., Lee K.E., Park Y.J., Yi K.H., Park D.J. (2016). Prognostic effects of TERT promoter mutations are enhanced by coexistence with BRAF or RAS mutations and strengthen the risk prediction by the ATA or TNM staging system in differentiated thyroid cancer patients. Cancer.

[12] Yip L., Nikiforova M.N., Yoo J.Y., McCoy K.L., Stang M.T., Armstrong M.J., Nicholson K.J., Ohori N.P., Coyne C., Hodak S.P. (2015). Tumor genotype determines phenotype and disease-related outcomes in thyroid cancer: A study of 1510 patients. Ann. Surg..

[13] Schumm M.A., Shu M.L., Hughes E.G., Nikiforov Y.E., Nikiforova M.N., Wald A.I., Lechner M.G., Tseng C.H., Sajed D.P., Wu J.X. (2023). Prognostic Value of Preoperative Molecular Testing and Implications for Initial Surgical Management in Thyroid Nodules Harboring Suspected (Bethesda V) or Known (Bethesda VI) Papillary Thyroid Cancer. JAMA Otolaryngol. Head Neck Surg..

[14] Yip L., Gooding W.E., Nikitski A., Wald A.I., Carty E., Karslioglu-French E., Seethala R.R., Zandberg D.P., Ferris R.L., Nikiforova M.N. (2021). Risk assessment for distant metastasis in differentiated thyroid cancer using molecular profiling: A matched case-control study. Cancer.

[15] Liu J.B., Baugh K.A., Ramonell K.M., McCoy K.L., Karslioglu-French E., Morariu E.M., Ohori N.P., Nikiforova M.N., Nikiforov Y.E., Carty S.E. (2023). Molecular Testing Predicts Incomplete Response to Initial Therapy in Differentiated Thyroid Carcinoma Without Lateral Neck or Distant Metastasis at Presentation: Retrospective Cohort Study. Thyroid.

[16] Zafon C., Gil J., Pérez-González B., Jordà M. (2019). DNA methylation in thyroid cancer. Endocr. Relat. Cancer.

[17] Rogucki M., Buczyńska A., Krętowski A.J., Popławska-Kita A. (2021). The Importance of miRNA in the Diagnosis and Prognosis of Papillary Thyroid Cancer. J. Clin. Med..

[18] Nieto H.R., Thornton C.E.M., Brookes K., Nobre de Menezes A., Fletcher A., Alshahrani M., Kocbiyik M., Sharma N., Boelaert K., Cazier J.B. (2022). Recurrence of Papillary Thyroid Cancer: A Systematic Appraisal of Risk Factors. J. Clin. Endocrinol. Metab..

[19] Titov S.E., Ivanov M.K., Demenkov P.S., Katanyan G.A., Kozorezova E.S., Malek A.V., Veryaskina Y.A., Zhimulev I.F. (2019). Combined quantitation of HMGA2 mRNA, microRNAs, and mitochondrial-DNA content enables the identification and typing of thyroid tumors in fine-needle aspiration smears. BMC Cancer.

[20] Ravi N., Yang M., Mylona N., Wennerberg J., Paulsson K. (2020). Global RNA Expression and DNA Methylation Patterns in Primary Anaplastic Thyroid Cancer. Cancers.

[21] Wojtas B., Pfeifer A., Oczko-Wojciechowska M., Krajewska J., Czarniecka A., Kukulska A., Eszlinger M., Musholt T., Stokowy T., Swierniak M. (2017). Gene Expression (mRNA) Markers for Differentiating between Malignant and Benign Follicular Thyroid Tumours. Int. J. Mol. Sci..

[22] Poma A.M., Giannini R., Piaggi P., Ugolini C., Materazzi G., Miccoli P., Vitti P., Basolo F. (2018). A six-gene panel to label follicular adenoma, low- and high-risk follicular thyroid carcinoma. Endocr. Connect..

[23] Mussazhanova Z., Shimamura M., Kurashige T., Ito M., Nakashima M., Nagayama Y. (2020). Causative role for defective expression of mitochondria-eating protein in accumulation of mitochondria in thyroid oncocytic cell tumors. Cancer Sci..

[24] Nan B.Y., Xiong G.F., Zhao Z.R., Gu X., Huang X.S. (2021). Comprehensive Identification of Potential Crucial Genes and miRNA-mRNA Regulatory Networks in Papillary Thyroid Cancer. Biomed Res. Int..

[25] Ma J., Han W., Lu K. (2021). Comprehensive Pan-Cancer Analysis and the Regulatory Mechanism of ASF1B, a Gene Associated With Thyroid Cancer Prognosis in the Tumor Micro-Environment. Front. Oncol..

[26] Zafereo M., McIver B., Vargas-Salas S., Domínguez J.M., Steward D.L., Holsinger F.C., Kandil E., Williams M., Cruz F., Loyola S. (2020). A Thyroid Genetic Classifier Correctly Predicts Benign Nodules with Indeterminate Cytology: Two Independent, Multicenter, Prospective Validation Trials. Thyroid.

[27] Kebebew E., Peng M., Reiff E., Duh Q.Y., Clark O.H., McMillan A. (2005). ECM1 and TMPRSS4 are diagnostic markers of malignant thyroid neoplasms and improve the accuracy of fine needle aspiration biopsy. Ann. Surg..

[28] Fryknäs M., Wickenberg-Bolin U., Göransson H., Gustafsson M.G., Foukakis T., Lee J.J., Landegren U., Höög A., Larsson C., Grimelius L. (2006). Molecular markers for discrimination of benign and malignant follicular thyroid tumors. Tumour Biol..

[29] Colombo C., Minna E., Gargiuli C., Muzza M., Dugo M., De Cecco L., Pogliaghi G., Tosi D., Bulfamante G., Greco A. (2020). The molecular and gene/miRNA expression profiles of radioiodine resistant papillary thyroid cancer. J. Exp. Clin. Cancer Res..

[30] Binabaj M.M., Soleimani A., Rahmani F., Avan A., Khazaei M., Fiuji H., Soleimanpour S., Ryzhikov M., Ferns G.A., Bahrami A. (2019). Prognostic value of high mobility group protein A2 (HMGA2) over-expression in cancer progression. Gene.

[31] Tavares C., Coelho M.J., Eloy C., Melo M., Gaspar da Rocha A., Pestana A., Batista R., Bueno Ferreira L., Rios E., Selmi-Ruby S. (2018). NIS expression in thyroid tumors, relation with prognosis clinicopathological and molecular features. Endocr. Connect..

[32] Chai L., Han D., Li J., Lv Z. (2018). The construction and analysis of gene co-expression network of differentially expressed genes identifies potential biomarkers in thyroid cancer. Transl. Cancer Res..

[33] Pozdeyev N., Gay L.M., Sokol E.S., Hartmaier R., Deaver K.E., Davis S., French J.D., Borre P.V., LaBarbera D.V., Tan A.C. (2018). Genetic Analysis of 779 Advanced Differentiated and Anaplastic Thyroid Cancers. Clin. Cancer Res..

[34] Feng J., Shen F., Cai W., Gan X., Deng X., Xu B. (2018). Survival of aggressive variants of papillary thyroid carcinoma in patients under 55 years old: A SEER population-based retrospective analysis. Endocrine.

[35] Baloch Z.W., Asa S.L., Barletta J.A., Ghossein R.A., Juhlin C.C., Jung C.K., LiVolsi V.A., Papotti M.G., Sobrinho-Simões M., Tallini G. (2022). Overview of the 2022 WHO Classification of Thyroid Neoplasms. Endocr. Pathol..

[36] Jung C.K., Jung S.H., Jeon S., Jeong Y.M., Kim Y., Lee S., Bae J.S., Chung Y.J. (2020). Risk Stratifcation Using a Novel Genetic Classifer Including PLEKHS1 Promoter Mutations for Diferentiated Thyroid Cancer with Distant Metastasis. Thyroid.

[37] Santiago K., Chen Wongworawat Y., Khan S. (2020). Differential MicroRNA-Signatures in Thyroid Cancer Subtypes. J. Oncol..

[38] Lukyanov S.A., Sergiyko S.V., Titov S.E., Reshetov I.V., Veryaskina Y.A., Vazhenin A.V., Gostimsky A.V., Ippolitov L.I., Rogova M.O. (2020). Stratification of papillary thyroid cancer relapse risk based on the results of molecular genetic studies. Head Neck Tumors.

[39] Titov S.E., Demenkov P.S., Lukyanov S.A., Sergiyko S.V., Katanyan G.A., Veryaskina Y.A., Ivanov M.K. (2020). Preoperative detection of malignancy in fine-needle aspiration cytology (FNAC) smears with indeterminate cytology (Bethesda III, IV) by a combined molecular classifier. J. Clin. Pathol..

[40] Livak K.J., Schmittgen T.D. (2001). Analysis of relative gene expression data using real-time quantitative PCR and the 2(-Delta Delta C(T)) Method. Methods.

[41] Chen C., Ridzon D.A., Broomer A.J., Zhou Z., Lee D.H., Nguyen J.T., Barbisin M., Xu N.L., Mahuvakar V.R., Andersen M.R. (2005). Real-time quantification of microRNAs by stem-loop RT-PCR. Nucleic Acids Res..

